# Asymmetric Cancer Hallmarks in Breast Tumors on Different Sides of the Body

**DOI:** 10.1371/journal.pone.0157416

**Published:** 2016-07-06

**Authors:** Emanuel M. Campoy, Sergio R. Laurito, María T. Branham, Guillermo Urrutia, Angela Mathison, Francisco Gago, Javier Orozco, Raul Urrutia, Luis S. Mayorga, María Roqué

**Affiliations:** 1 IHEM-CONICET, Mendoza, Argentina; 2 Faculty of Medical Sciences, National University of Cuyo, Mendoza, Argentina; 3 Faculty of Exact Sciences, National University of Cuyo, Mendoza, Argentina; 4 Epigenetics and Chromatin Dynamics Laboratory, GI Research Unit, Division of Gastroenterology and Hepatology, Epigenomics Translational Program, Center for Individualized Medicine, Mayo Clinic, Rochester, Minnesota, United States of America; 5 Gineco-Mamario Institute, San Lorenzo, Mendoza, Argentina; 6 Italian Hospital of Mendoza, Mendoza, Argentina; University of Campinas, BRAZIL

## Abstract

During the last decades it has been established that breast cancer arises through the accumulation of genetic and epigenetic alterations in different cancer related genes. These alterations confer the tumor oncogenic abilities, which can be resumed as cancer hallmarks (CH). The purpose of this study was to establish the methylation profile of CpG sites located in cancer genes in breast tumors so as to infer their potential impact on 6 CH: i.e. sustained proliferative signaling, evasion of growth suppressors, resistance to cell death, induction of angiogenesis, genome instability and invasion and metastasis. For 51 breast carcinomas, MS-MLPA derived-methylation profiles of 81 CpG sites were converted into 6 CH profiles. CH profiles distribution was tested by different statistical methods and correlated with clinical-pathological data. Unsupervised Hierarchical Cluster Analysis revealed that CH profiles segregate in two main groups (bootstrapping 90–100%), which correlate with breast laterality (p = 0.05). For validating these observations, gene expression data was obtained by RealTime-PCR in a different cohort of 25 tumors and converted into CH profiles. This analyses confirmed the same clustering and a tendency of association with breast laterality (p = 0.15). *In silico* analyses on gene expression data from TCGA Breast dataset from left and right breast tumors showed that they differed significantly when data was previously converted into CH profiles (p = 0.033). We show here for the first time, that breast carcinomas arising on different sides of the body present differential cancer traits inferred from methylation and expression profiles. Our results indicate that by converting methylation or expression profiles in terms of Cancer Hallmarks, it would allow to uncover veiled associations with clinical features. These results contribute with a new finding to the better understanding of breast tumor behavior, and can moreover serve as proof of principle for other bilateral cancers like lung, testes or kidney.

## Introduction

During the last two decades, it has been established that cancer is in essence a genetic disease. Over the past years, the cancer genome has been studied by different molecular strategies, revealing alterations in many cancer related genes. And several studies have shown that the majority of human tumors carry mutations in a subset of genes, composed of 30 to 60 different affected loci [1]. Inferences drown from these observations indicate that cancers as diseases, are caused by the emergence of distinct “genomic landscapes” composed of a combination of these mutations. A “cancer genomic landscape” for a particular tumor is known to be composed by few genes called “drivers” (frequently found altered across many cancers), and many more genes called “passengers” (seldom found altered across cancers) [1]. Consequently, these discoveries have guided the development of targeted therapies against single driver genes, including gefitinib and erlotinib for non-small-cell lung cancer patients with EGFR mutations [2], panitumumab and cetuximab for metastatic colon cancer with amplified expression of EGFR [3], vemurafenib for patients with melanomas carrying BRAF mutations [4], and crizotinib for lung cancer patients carrying EML4-ALK translocations [5]. However, in spite of all the remarkable advances, new information strongly suggests that therapeutically targeting single driver genes is not a reliable strategy for the long term treatment of cancers [1,2], since cancer traits appear to be better described when genomic data is interpreted as a network of combined functional pathways. “Cancer genomic landscapes” functioning as interconnected pathways may help to explain the existence of “Cancer Hallmarks” (CH) proposed by Hanahan and Weinberg in 2000 [6]. They propose that the cancer phenotype is characterized by a few biological capabilities acquired during a multistep process of carcinogenesis, i.e. sustained proliferative signaling, evasion of growth suppressors, resistance to cell death, enabled replicative immortality, induction of angiogenesis, and activation of invasion and metastasis. Emerging evidence also indicates that the reprogramming of energy metabolism and evasion of immune destruction could be considered two new cancer hallmarks [7]. These six to eight cancer hallmarks provide an organizing principle for understanding the complexity and diversity of neoplastic diseases.

Moreover, in addition to genetics, epigenetics, a mode of inheritance that is brought about independently of genetic sequences, offers a complementary paradigm for better understanding the behavior of cancer. Epigenomics have revealed that cancers can also be caused and maintained by inherited alterations of gene expression networks. Thus, it has become evident that CHs are acquired by a successive accumulation of both genetic and epigenetic alterations, which are transmitted to subsequent cellular generations. These genetic and epigenetic alterations occur at many different genomic regions [1] but their effect still converges in less than ten tumor capabilities [7], which implies that the gene functions involved in tumorigenesis are function of the state of the mutated network [8]. Since the study of epigenetic alterations lags significantly behind those performed in genetics, in the current study, we sought to better understand the role of epigenetic alterations that occur during breast cancer and unraveling their potential application in the diagnosis and prognosis of this disease. Although many genes have been encountered aberrantly methylated in breast tumors and associated to worst prognosis [9–13], the breast cancer field still lacks information for unifying treatment decisions based on epigenetic markers. Epigenetic alterations in breast cancer consist of chromatin modifications, including DNA methylation and histone modifications, and regulatory small and large RNA molecules, contributing all in different ways to regulate gene expression. The best understood among them is DNA hypermethylation, which occurs within CpG islands (CGIs), (particularly in the promoter and first exon regions), blocking the binding sites of transcription factors and therefore strongly associated with gene repression [14].

The purpose of this research work was to establish the methylation profile of CpG sites located in cancer related genes (mainly tumor suppressor genes and DNA repair genes) in fresh human breast tumors so as to infer their potential impact on Cancer Hallmarks. Our results indicate that by converting methylation profiles in terms of Cancer Hallmarks, it would allow to better interpret this type of epigenetic data in a holistic manner and uncover veiled associations with clinical features. Therefore, the mechanistic importance and biomedical relevance of these results are discussed.

## Materials and Methods

### Patients and tumor samples

Patients (113) were enrolled in the current study after obtaining their written informed consent based on the scientific and ethical principles of the World Medical Association’s Declaration of Helsinki (Table 1). Ethical approval of the project and the written informed consent was obtained from the Ethics Committee of the Faculty of Medical Sciences, from the National University of Cuyo, Mendoza, Argentina. One hundred thirteen tumors were collected for methylation analyses, to establish their methylation frequencies in the sample cohort. Seventy-six presented complete clinical-pathological data, of which a cohort of 51 was used for further conversion to CH profiles and posterior statistical analyses and a different cohort of 25 tumors was used to perform Real-Time PCR experiments. We generated a database containing the clinical-pathological information of each patient, in addition to the DNA methylation and gene expression profiles of the respective tumors (S1 File). The clinical-pathological features were assessed by the same pathologist and all the patients were treated in either the Gineco-Mamario Institute or the Italian Hospital of Mendoza, Argentina.

**Table 1:** Patient and Tumor characteristics.

	Total Number	Methylation Analysis	Expression Analysis
Patients	113		
Tumors for methylation analyses	113		
Patients with complete clinical data	74	51	23
Age (years)*			
≤40	10	5	5
>40	60	45	15
Unknown	4	1	3
Tumors with complete anatomo-pathological data	76	51	25
Tumor Types			
DCIS	2	1	1
IDC	67	45	22
ILC	4	3	1
Others	3	2	1
Axillar Lymph Node Status			
Positive	36	27	9
Negative	40	24	16
Tumor Grade			
I	9	8	1
II	27	21	6
III	40	22	18
Disease Stage			
1	30	20	10
2A	19	14	5
2B	9	5	4
3A	13	10	3
3B	1	0	1
3C	3	2	1
Unknown	1	0	1
Breast Laterality			
Right Side	42	29	13
Left Side	34	22	12
Molecular Subtypes			
Luminal/HER2	55	32	23
TN	21	19	2

* Mean age: 56.48 years (SEM = 1.70)

DCIS: Ductal Carcinoma In Sit; IDC: Invasive Ductal Carcinoma, ILC: Invasive Lobular Carcinoma

HER2: Human Epidermal Growth Factor Receptor 2; TN: Triple Negative

### DNA extraction

Fresh tissues were frozen at -80°C and broken with a frozen mortar. The homogenate was collected and suspended in T_10_E buffer (10mM Tris/HCl and 1mM EDTA). All samples were stored for at least 24 hours at -20°C and DNA was collected as previously described [15]. Briefly, homogenate from tumor tissues was suspended in 3ml of Cetyl Trimethylammonium Bromide (CTAB) solution (2g/l CTAB, Sigma Aldrich, Bavaria Germany, 100mM Tris/HCl, 20mM EDTA and 2% 2-mercaptoethanol) and incubated at 60°C during 4 hours for membrane lysis. Afterwards, 3ml of chloroform-isoamylic solution (24:1) was added and centrifuged at 3000 rpm for 5 minutes. Aqueous phase was collected into a new tube and mixed with 9ml ice-cold 100% ethanol. Precipitated DNA was dissolved in T_10_E buffer and stored at -20°C.

### Methylation profiles determination by Methyl specific-Multiplex Ligation-dependent Probe Amplification assay (MS-MLPA)

To assess the methylation status of 96 CpG sites located within 54 cancer related genes, the MS-MLPA kits ME001, ME002, ME003 and ME011 were used (MRC-Holland, Amsterdam, The Netherlands, www.mlpa.com). The MS-MLPA assays were performed basically according to manufacturer’s recommendations, introducing subtle modifications (i.e. extended restriction enzyme incubation time, separated ligation and restriction steps) [12]. The fluorescent-labeled PCR products were separated by capillary electrophoresis in an ABI-3130 sequencer (Applied Biosystems, Foster City, CA, USA) and analyzed by GeneMarker v1.75 software (Softgenetics, State College, PA, USA). This analysis normalizes the data by dividing the peak area of a single probe by the peak areas of control probes. Subsequently, the normalized peaks from the samples were divided by the normalized peaks from controls to obtain the Methylation Dosage Ratio (MDR). A CpG site was considered to be methylated when the MDR observed between sample and control was superior to the cut-off threshold of 8% [15,16]. Afterwards, DNA methylation data was dichotomized in unmethylated and methylated status.

### Gene expression analyses by Real-Time Polymerase Chain Reaction

RNA was extracted from fresh tumors with Trizol Reagent (Life Technologies, USA). Five μg of total RNA was used for first strand synthesis of cDNA by using M-MLV retro-transcriptase (Promega, USA) and Random Hexamers (Roche, USA) primers. The reverse transcription reaction was carried out during 60 minutes at 37°C according to manufacturer´s instructions. One hundred ng of cDNA were used to perform Real-Time PCR using specific primers for 32 cancer related genes (Table 2) and GAPDH, TBP and HPRT1 as housekeeping genes in a CFX96 thermocycler (Bio-Rad Laboratories, USA). Detection of PCR product was carried out using the specific DNA dye SYBR Green (Bio-Rad, USA). The amplification program consisted of 45 cycles of 15 seconds at 95°C and 60 seconds at 60°C, followed by a final melting curve step. The specificity of the PCR products was assessed by melting curve analysis. Relative expression normalization of genes of interest was carried out using the housekeeping genes GAPDH, TBP and HPRT1 (gene expression as endogenous reference control by the ddCt method). Cycle Threshold (Ct) and Efficiency (EAmp) values were calculated by means of Bio-Rad CFX Manager 3.0 (Bio-Rad Laboratories, USA). After relativizing gene expression to the housekeeping genes, data of each gene was expressed in percentages considering as 100% the highest expression value among all tumors. Subsequently, values were inverted by applying the equation “100 –expression value”. This was necessary to multiply afterwards the inverted data by the “Translation Matrix” in order to create the CH profile matrix for further clustering analyses (see Results Section).

**Table 2:** CpG location, genes and methylation frequencies in 113 breast tumors.

CpG sites respect to ATG	Gene Name (HGDB)	Chromosome Location	Methylation Frequency (%)	CpG sites respect to ATG	Gene Name (HGDB)	Chromosome Location	Methylation Frequency (%)
72 bp to exon 2	APC	5q22	43.68	13 bp before	MLH1	3p22.2	4.42
4457 bp before	ATM	11q23	3.6	206 bp after	MLH1	3p22.2	7.69
4658 bp before	ATM	11q23	4.6	85–86 bp in	MLH3	14q24.3	1.54
870 bp before	BCL2	18q21.3	4.42	269 bp before	MSH2	14q24.3	8.85
1211 bp before	BRCA1	17q21.31	10.34	193 bp before	MSH2	14q24.3	8.85
1321 bp before	BRCA1	17q21.3	7.21	124 bp after	MSH2	2p21	8.85
852 bp before	BRCA2	13q12.3	6.9	485 bp before	MSH3	5q14.1	35.38
771 bp before	BRCA2	13q12.3	9.73	317 bp before	MSH6	2p16.3	5.75
157 bp after	CACNA1A	19p13.2	31.53	126 bp before	MSH6	2p16.3	1.54
32 bp before	CACNA1G	17q21.33	8.18	32 bp before	MSH6	2p16.3	7.69
8560 bp before	CASP8	2q33.2	8.14	661 bp before	PAX5	9p13	60.92
1168 bp before	CCND2	12p13.3	72.41	49 bp before	PAX6	11p13	41.38
1358 bp before	CCND2	12p13.3	54.02	306 bp before	PM\	7p22.1	9.91
17 bp before	CD44	11p12	18.92	62 bp before	PMS2	7p22.1	12.31
411 bp before	CD44	11p12	15.04	40 bp after	PMS2	7p22.1	3.19
42 bp before	CDH13	16q23.3	28.32	1837 bp before	PTEN	10q23.3	4.42
157 bp before	CDKN1B	12p13.2	3.08	1110 bp before	PTEN	10q23.3	12.61
997 bp before	CDKN2A	9p21	0.9	651 bp before	RARB	3p24.2	16.22
31 bp after	CDKN2A	9p21.3	7.21	824 bp before	RARB	3p24.2	26.44
110 bp before	CDKN2B	9p21	17.12	888 bp before	RARB	3p24.2	8.05
714 bp before	DAPK1	9q22	18.02	141 bp before	RASSF1	3p21.3	67.57
366437 bp after	DLC1	8p22	20.69	79 bp before	RASSF1	3p21.3	72.97
366993 bp after	DLC1	8p22	6.19	520 bp before	RB	13q14.2	6.15
163 bp after	ESR1	6q25.1	20.72	323 bp before	RB1	13q14.2	11.49
658 bp before	GATA5	20q13.3	21.84	18 bp before	RUNX3	1p36.11	24.78
103 bp after	GSTP1	11q13	29.73	232 bp before	SCGB3A1	5q35	66.67
245 bp before	GSTP1	11q13	14.16	17 bp before	SCGB3A1	5q35	38.05
953 bp before	ID4	6p22.3	57.52	42 bp before	SFRP4	7p14.1	20.69
319 bp before	ID4	6p22.3	27.59	275 bp before	SFRP5	7p14.1	21.05
305 bp before	IGSF4	11q23	16.22	316 bp before	SFRP5	10q24.1	40.71
72 bp before	IGSF4	11q23	32.18	82 bp after	SRFP4	10q24.1	18.6
432 bp before	MGMT	10q26.3	3.08	834 bp before	THBS1	15q15	9.2
346 bp before	MGMT	10q26.3	3.08	172 bp before	TIMP3	22q12.3	13.51
93 bp before	MGMT	10q26.3	20.35	300 bp before	TIMP3	22q12.13	4.42
151 bp after	MGMT	10q26.3	3.08	50 bp after	TIMP3	22q12.13	8.11
382 bp before	MGMT	10q26.3	1.54	10905 bp before	TP53	17p13.1	6.19
233 bp after	MLH1	3p22.3	0	29790 bp before	TP73	1p36.32	2.7
659 bp before	MLH1	3p22.3	1.8	29551 bp before	TP73	1p36.3	45.05
518 bp before	MLH1	3p22.1	0	220 bp after	TWIST1	7p21.2	10.77
383 bp before	MLH1	3p22.2	1.77	412 bp before	WT1	11p13	89.38
246 bp before	MLH1	3p22.1	12.31				

### Statistical analyses

To determine whether the methylations in the included genes were independent events, we generated 1000 hypothetic tumors and compared the distribution of CHs (derived from methylation profiles) between experimental and hypothetical tumors. For this, Kolmogorov-Smirnov test was applied using software KyPlot 3.0. To test tumor distribution based on CH profiles, Unsupervised Hierarchical Cluster Analysis using the software MultiExperiment ViewerMeV v4.6 (TM 4 group, Dana Farber Cancer Institute, Boston, MA, USA) was used. For this last analysis, we considered 90–100% bootstrapped clusters. To asses which CH contributed to the clustering, Principal Component Analyses (PCA) and Multiple Regression Analyses of clusters vs. CH were performed applying software InfoStat v.2014 (Grupo InfoStat, FCA, Universidad Nacional de Córdoba, Argentina). For association studies between clinical-pathological variables and clusters, Simple Regression Analyses were performed using the software SPSS v17 (SPSS Inc, Chicago, IL, USA). Multiple Regression Analyses on partitioned samples were performed to determine associations between CH and clinical-pathological variables by the software InfoStat v.2014 (Grupo InfoStat, FCA, Universidad Nacional de Córdoba, Argentina).

## Results

### Conversion of MLPA-derived Methylation Data into Functional Cancer Hallmarks

We applied the Methyl Specific Multiplex Ligation-dependent Probe Amplification assay (MS-MLPA) to determine the methylation status of 96 CpG sites located within 54 cancer related genes of 113 breast carcinomas. It is important to take in account that previous studies of our group revealed that none of these CpG sites is methylated in normal breast tissue obtained from surgical margins or in circulating leucocytes [12,15]. Therefore, it becomes reasonable to only focus on aberrant methylation events since interference of normal cells is discarded. The methylation frequency of each CpG site was determined on the complete tumor cohort. Based on the obtained frequency, in order to decrease the ambiguity given by the inclusion of poorly methylated sites, we excluded CpG sites methylated in less than 10% of the samples. This criterion reduced the number of CpG sites from 96 to 81, located within the promoter of 43 distinct cancer related genes (Table 2). Thus, the 113 tumors served to determine methylation frequencies and establish the 43 crucial genes to continue the study.

Subsequently, the conversion of methylation data into CH profiles had to be performed. We decided to perform this only for 51 tumors of which we had complete clinical-pathological information. For this purpose, we organized the methylation data of the 51 tumors into a matrix which included for each tumor, the methylation status of each CpG site, determined by a dichotomized criterion (unmethylated and methylated).

**Fig 1:**
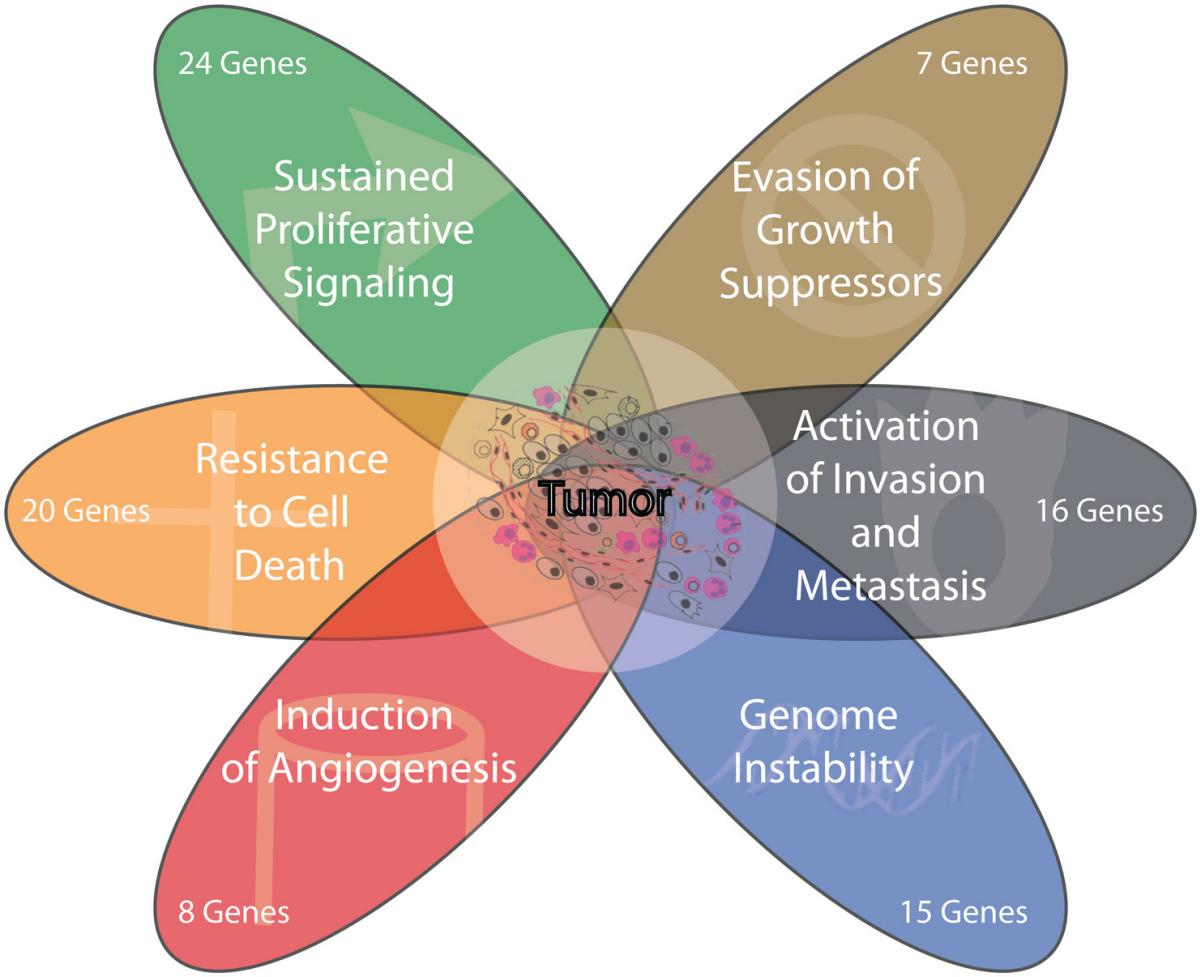
Hallmarks of Cancer selected for the study. Five of the six cancer hallmarks proposed by Hanahan and Weinberg were selected for this study: i.e. Sustained proliferative signaling (CH1), Evasion of growth suppressors (CH2), Resistance to cell death (CH3), Induction of angiogenesis (CH4) and Activation of invasion and metastasis (CH6) The Hanahan-Weinberg hallmark “Enabled replicative immortality” is replaced in this study by “Genome instability” (CH5) as an alternative hallmark. The number of genes participating in the CH are shown each ellipse.

For the conversion into cancer hallmarks, we selected 5 of the 6 cancer hallmarks proposed by Hanahan and Weinberg, i.e. sustained proliferative signaling (CH1), evasion of growth suppressors (CH2), resistance to cell death (CH3), induction of angiogenesis (CH4) as well as activation of invasion and metastasis (CH6). We did not include the Hanahan-Weinberg hallmark “enabled replicative immortality” because our study lacked of genes related to this feature. Instead, we decided to consider the alternative hallmark “genome instability” (CH5) (Fig 1), given many included genes (15 of 43) had a role in DNA repair.

By data mining studies, including literature and databases from NCBI (http://www.ncbi.nlm.nih.gov/), Uniprot (http://www.uniprot.org/), Nextprot (http://www.nextprot.org/) and DAVID Bioinformatics Resources 6.7 (https://david.ncifcrf.gov/), the influence of the 43 genes on the 6 selected CH was defined. For this purpose, we developed a priory a gene-to-function Participation Index (PI), with values ranking from 0 (no participation) to 3 (high participation), subsequently adjusted by a correction coefficient (ranked from 0 to 1) depending on data reliability, potentially contradictory data, or ambiguous interpretation to derive an Adjusted Participation Index (API). It is worth to remember that the participation of a gene in a specific CH was inferred considering the scenario in which the gene was methylated. These efforts led us to establish, that among the 43 genes, 24 of them associated to functions compatible with CH1, 7 genes in CH2, 20 genes to CH3, 8 genes to CH4, 15 genes to CH5 and 16 genes to CH6. Thus, from this data we inferred that all the CHs were related to at least 7 genes and that a single gene could be included in more than one CH. So, the API values were established for the 43 genes, generating what we called the “translation matrix”.

**Fig 2:**
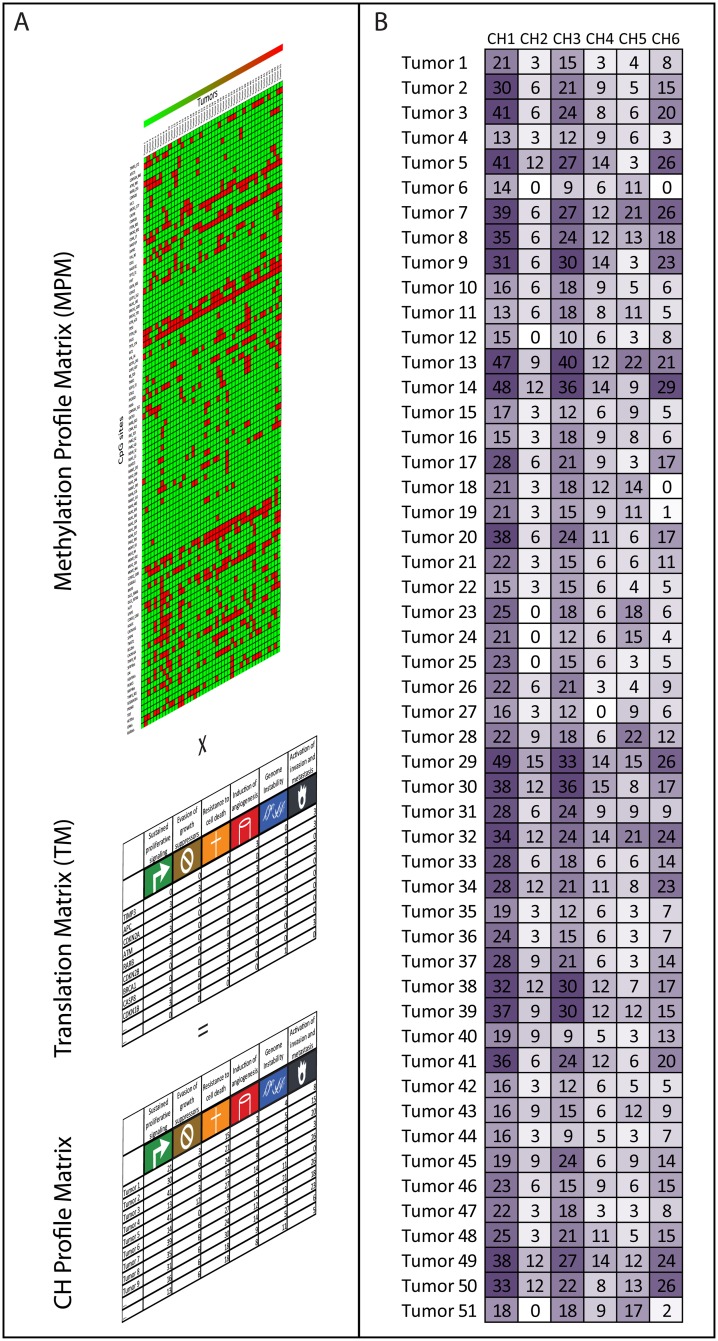
Translation of Methylation profile to Cancer Hallmark profile. (A) The scheme describes how the MLPA-derived methylation data was converted into CH profiles, though a translation matrix. A multiplication operation was performed on two matrices: the Methylation Profile Matrix (MPM) and the Translation Matrix (TM). The MPM holds information of 51 CpGs located in 43 genes the MPM, for 51 tumors with complete clinical-pathological information. Green boxes represent the un-methylated status and red boxes the methylated status. The TM contains the Adjusted Participation Index (API) which expresses in a rank from 0 to 3, the influence of each of the 43 genes on the 6 studied Cancer Hallmarks (CH). This multiplication of both results in a CH Profile Matrix, which represents for each tumor, the values with which each CH is enhanced. The higher the values of the CH matrix, the more the methylation events have contributed to acquire specific CHs. (B) Results of the matrices multiplication operation performed for the 51 studied tumors. Each tumor (rows) presents a specific CH profile. The CH values are represented in a colored grey gradient (light grey are lower values; dark grey represent higher values).

Now, by using this “translation matrix” the conversion of methylation data into the CH profiles had to be performed. By multiplying the methylation data and the “translation matrix”, we derived a CH Matrix (CHM) (Fig 2A), which shows the tumor traits now at a defined set of Hallmarks level. The higher the CHM values obtained, the stronger the magnitude of methylated derived cancer features. This data modeling approach considers that a given CH was supposed to be acquired if at least one of its component genes was methylated. Besides, some genes are members of multiple CHs, e.g. TIMP3 whose methylation is associated with CH4 and CH6, or ATM which is enrolled in CH1, CH3, CH5 and CH6 (S1 Fig). If such a gene was methylated, all hallmarks in which the gene had participation were considered to be enhanced.

Fig 2B shows the results obtained by converting MLPA-derived methylation data of 51 tumors into CH profiles. At first sight it can be observed that most of the tumors displayed CH1 (sustained proliferative signaling) and CH3 (resistance to cell death) as their strongest capabilities.

### Statistical Evidence Supports a Non Random Distribution of CH profiles in Breast Tumors

**Fig 3:**
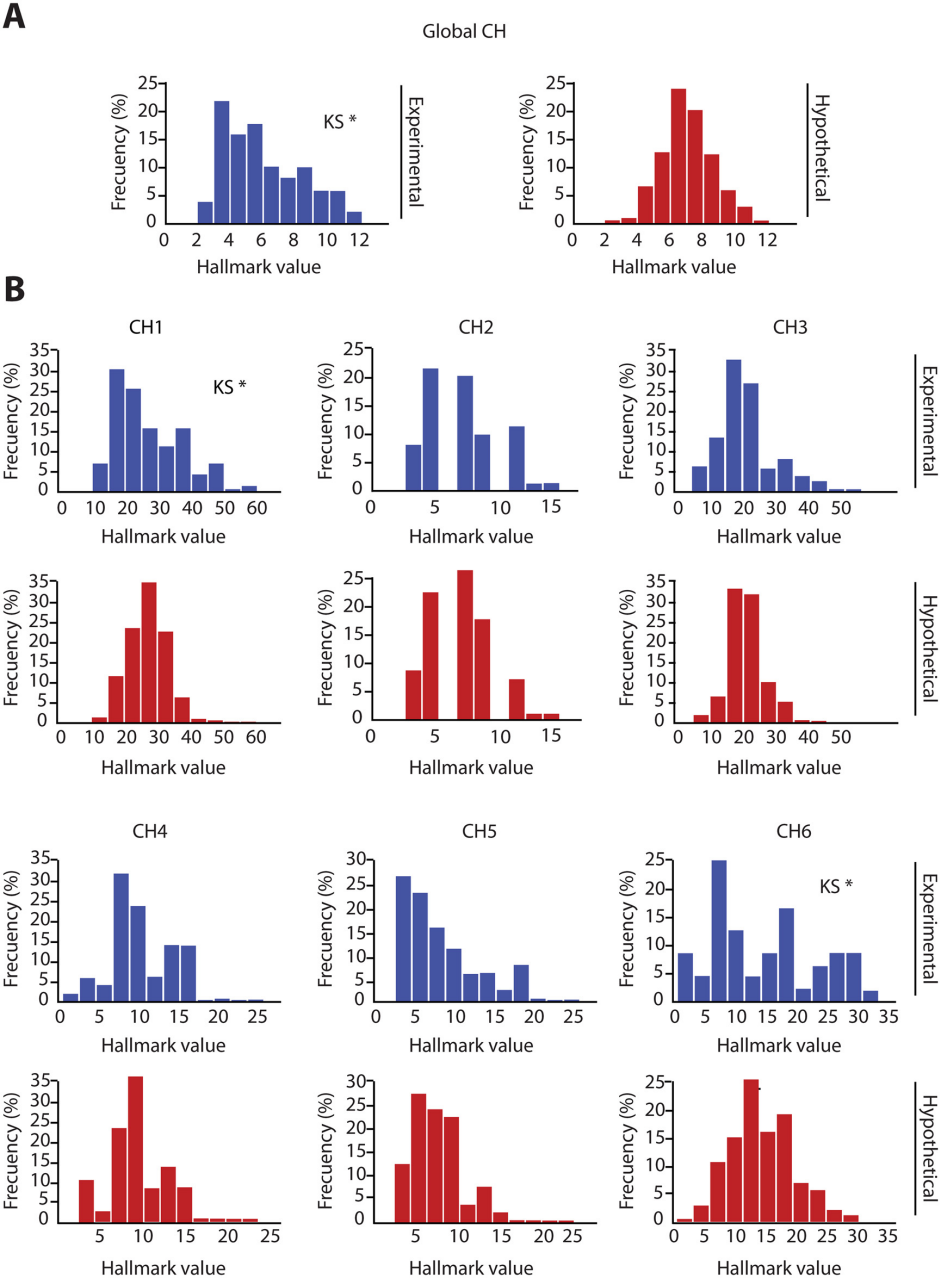
Cancer Hallmark profiles are not randomly distributed in experimental tumors. Histograms presenting the distribution of CH values in experimental vs artificial (hypothetical) tumors. (A) By summing the values of the 6 CHs in each tumor, a Global CH value was obtained. Comparisons of the histograms representing the distribution of the Global CH values of experimental vs hypothetical tumors revealed a significant difference (Kolmogorov Smirnov test (KS)*, p<0.05). (B) By performing the comparison of the distribution of the single CH values of experimental vs hypothetical tumors, a significant difference was detected for CH1 and CH6 (Kolmogorov Smirnov test (KS*), p<0.05). These differences are suggesting that the CH profiles in experimental tumors are not generated by random accumulation of CpG methylations.

From Fig 2B, we could conclude that the tumors were not similar. We speculated that CpG methylations are not independent events but rather are probably coordinated to affect the CH capabilities of the tumors in specific manners. To test these possibilities, we generated 1000 hypothetic tumors incorporating methylations of cancer related genes with a probability equal to the experimental methylation frequency. The purpose was to compare the observations of CHs in fresh (experimental) tumors, with randomly acquired CHs in artificial (hypothetical) tumors. For example, suppose the CpG site 1 presented a 15% frequency of methylation among experimental tumors. To create a hypothetical tumor, the methylation status of CpG site 1 was decided by generating a random number between 0 and 1 and considering the site methylated when the number was less than 0.15. This was performed for each of the 81 CpGs (considering the methylation frequency of each site) in 1000 artificial tumors. Afterwards, the methylation profiles were converted into CH profiles. With the CH values obtained by experimental and artificial tumors, we generated frequency histograms of the 6 CHs and compared whether they presented differences in a single or global way. We detected a significant difference for the global CH index (which resulted by summing the normalized single CH of each tumor) (Fig 3A) and for CH1 and CH6 (Fig 3B). These differences were suggesting that the CH profiles in experimental tumors were not generated by random accumulation of CpG methylations. If so, we speculated that the experimental tumors could be grouped depending on the CH acquisition. To test this, we performed Unsupervised Hierarchical Cluster Analyses of the CH profiles for the 51 experimental tumors.

**Fig 4:**
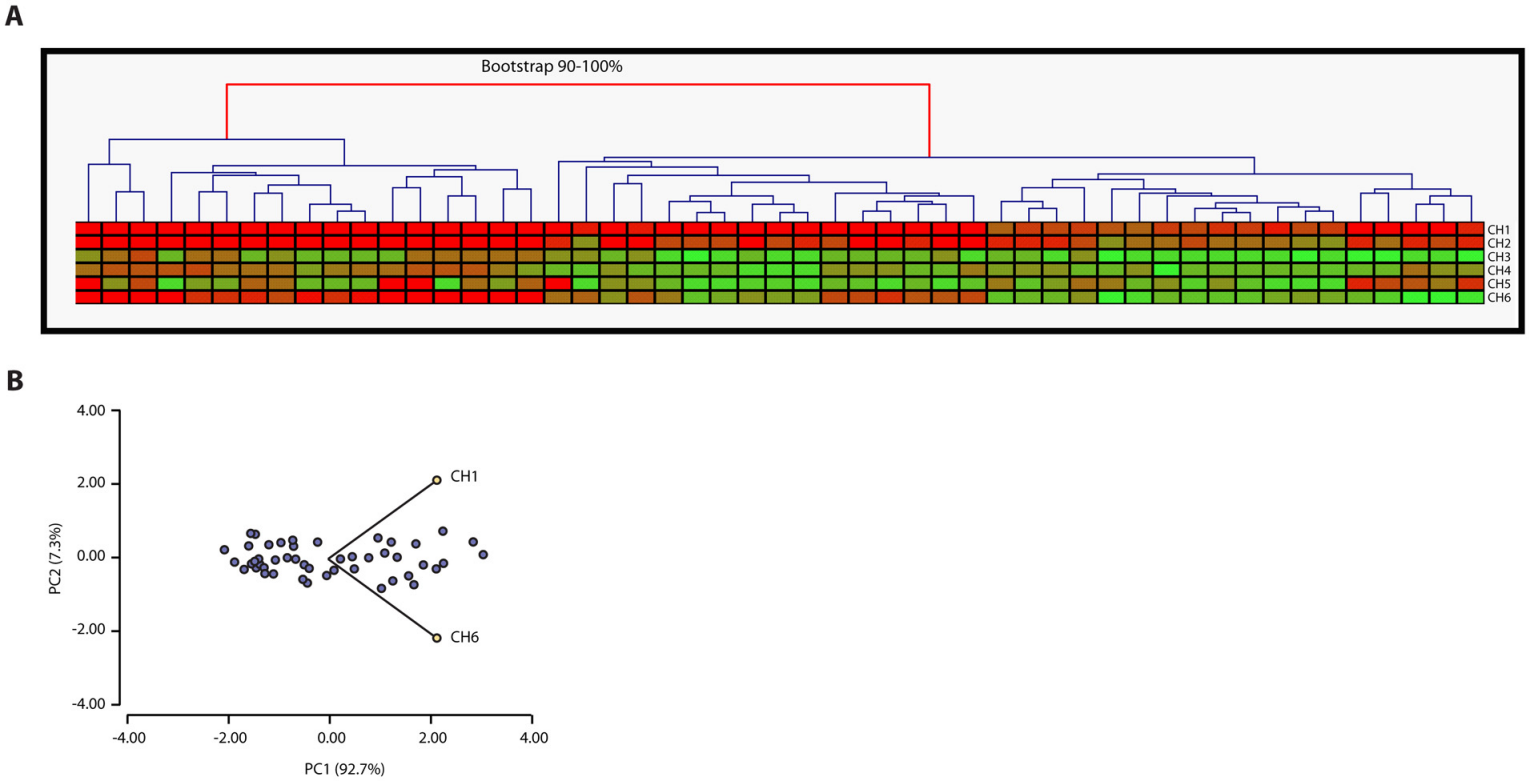
CH1 and CH6 define two tumor clusters. Clustering of CH profiles of 51 tumors. (A) Tumors are represented in columns, CHs in rows. A color gradient from green to red is used to represent low to high values of CHs (from 0 to 49). Unsupervised Hierarchical Cluster Analysis was performed by the software MultiExperiment Viewer MeV v4.6. Even though many clusters are formed, only two groups were established for bootstrapping 90–100% (shown as red arm). (B) Principal component analysis performed by software InfoStat v.2014 shows that 92.7% of the grouping is predictable by CH1 and CH6.

The test gave rise to two significant groups, whose robustness was determined by a 90–100% bootstrapped confidence interval (based on 100 iterations, average linkage and Manhattan distance metric) (Fig 4A). This was revealing that the tumors tended to cluster based on CH acquisition, which was in line with the fact that the acquisition was not at random. In order to understand more deeply how this grouping was occurring, we decided to investigate whether any of the 6 CHs had a main role in the group formation, so we performed a Principal Component Analysis (PCA) of the data. This analysis calculates the power of each CH (or Component) to predict the groups; the higher the power, the more principal is the CH for the group formation. This test showed that the clustering could be predicted in 92.7% of cases by CH1 and CH6 (Fig 4B). Another way of testing prediction value of the CHs is by Multiple lineal Regression Analysis of clusters vs. CHs. This test confirmed that CH1 (p<0.01), CH4 (p = 0.05) and CH6 (p = 0.11) were the best predictors of this grouping (adjusted R^2^ = 0.72).

On the contrary, artificial tumors did not reveal the same behavior, as PCA analyses performed on artificial tumors showed statistically different values (76.9% prediction power for CH1 and CH6) as compared with the experimental tumors. So, taken all together we were seeing that the experimental tumors were tending to cluster in two groups based on their CH profiles and that CH1 and CH6 were principal actors in this differentiation.

Interestingly, this was observable only at a CH level and not at methylation level data. When we performed Unsupervised Hierarchical Cluster Analysis on methylation profiles (instead of CH profiles), no clusters for 90–100% bootstrapped confidence intervals did appear. This was confirming that the conversion of MLPA-derived methylation data into functional CH profiles had been worth since analyses at hallmark-levels were revealing more information about the tumors.

### Cancer Hallmarks Profiles of Experimental Tumors More Robustly Associate to the Single Clinical-Pathological Feature of Breast Laterality (BL)

The next question which arose was: do the two tumor groups generated by CH profiles share clinical features? To address this question, we evaluated the association between the clusters and the clinical-pathological variables listed in Table 1, i.e. age, axillar lymph node status, tumor stage, disease stage, breast laterality and molecular subtype. Surprisingly, by Regression Analysis, only the variable *breast laterality* (BL) showed significant association with the tumor groups (p = 0.05). Remarkably, this was not observable when testing correlation between single methylation profiles with BL, supporting again the concept that hallmark-level analyses were more informative. So, the association of CH profiles with BL was suggesting us the hypothesis that tumors from different breast sides presented distinctive cancer hallmarks (breast laterality hypothesis, BLH).

**Fig 5:**
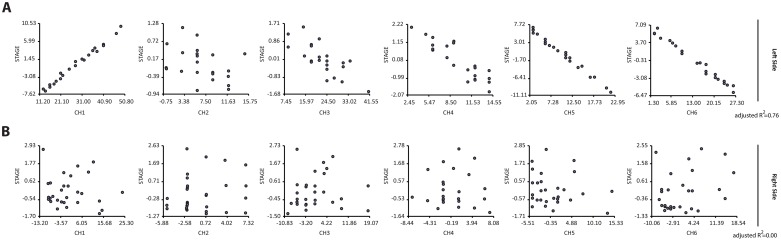
Tumors from left-right breast sides are differentially predicted by cancer hallmarks. Regression analyses of CHs vs tumor stage in left and right sided tumors. (A) CHs of left sided lesions were better predictors of the tumor stage, revealing that CH1 increased with tumor stage while CH4, CH5 and CH6 decreased (adjusted R^2^ = 0.76). (B) CHs of right sided tumors have no predictable value for tumor stages (adjusted R^2^ = 0.00), supporting thereby the conclusion that the behavior of CHs differs in tumors of different sides.

We were aware that the general understanding is that the breast tumor would be randomly located and would present similar features independently from the side. Consequently, to deepen the analysis, we partitioned the data in left and right and performed Multiple Regression tests by correlating the tumors with both the CHs and clinical-pathological variables. Indeed, in support of the laterality hypothesis stated above, this approach showed that the results differed according to breast side. In left sided lesions, CHs were better predictors of the tumor stage (adjusted R^2^ = 0.76), revealing that CH1 increased with tumor stage while CH4, CH5 and CH6 decreased (Fig 5A). This was not observable in right sided tumors, where the adjusted R^2^ was 0.00 for tumor stage (Fig 5B), supporting thereby the conclusion that the behavior of CHs is different in tumors of different sides.

We expanded our analyses in order to assess the possibility of confounding factors. In order to discard any influence of interfering variables, we compared the number of tumor types, axillar lymph nodes status, tumor grades, disease stages and receptor expression levels in left vs right tumors and detected no significant differences. By this we were able to exclude any interference of other clinical-pathological variables. Therefore, based on these evidences, we are able to propose that CHs inferred from methylation data are acquired in distinct ways according to the breast side on which the tumor develops.

### Expression Profiles Also Associate to the Single Clinical-Pathological Feature of Breast Laterality

To continue challenging our BL hypothesis, we thought to test a new cohort of tumors from a different perspective. We decided to validate the results thus far generated through the analysis of gene expression levels instead of methylation statuses, in a different tumor cohort (Table 1). The rationality of this was that even though not all methylation events included in our study would have the same effect on gene expression, if the associations inferred from methylation data were robust, we would be able to detect them also inferring from expression data. For this purpose, we performed Real-Time PCR analyses on 32 (Table 3) of the 43 genes on a new cohort of 25 breast carcinomas. The expression data was normalized to the average of 3 housekeeping genes and organized in an expression matrix which contained the information of the 25 new tumors. In a manner which is similar to the approach used above to convert methylation into CH level information, we converted now the expression matrix into CH profiles, through the “translation matrix” (shown in Fig 2A). Accordingly, we obtained a CH profile matrix, which allows drawing inferences from expression information rather than from methylation profiles. Notice that in contrast to the methylation results, the expression data appears to be inversely related to the CH profiles. Thereby, we inverted the values for further clustering analyses.

**Table 3:** Genes included in the expression analyses.

Gene Name (HGDB)	Chromosome Location
APC	5q22
ATM	11q23
BRCA1	17q21.3
BRCA2	13q12.3
CCND2	12p13.3
CD44	11p12
CDH13	16q23.3
CDKN1B	12p13.2
CDKN2A	9p21
CDKN2B	9p21
DAPK1	9q22
DLC1	8p22
ESR1	6q25.1
GSTP1	11q13
ID4	6p22.3
MGMT	10q26.3
MLH1	3p22.3
MSH2	2p21
MSH6	2p16.3
PAX6	11p13
PMS2	7p22.1
PTEN	10q23.3
RARB	3p24.2
RASSF1	3p21.3
RB1	13q14.2
SCGB3A1	5q35
SFRP4	7p14.1
THBS1	15q15
TP53	17p13.1
TP73	1p36.32
TWIST1	7p21.2
WT1	11p13

**Fig 6:**
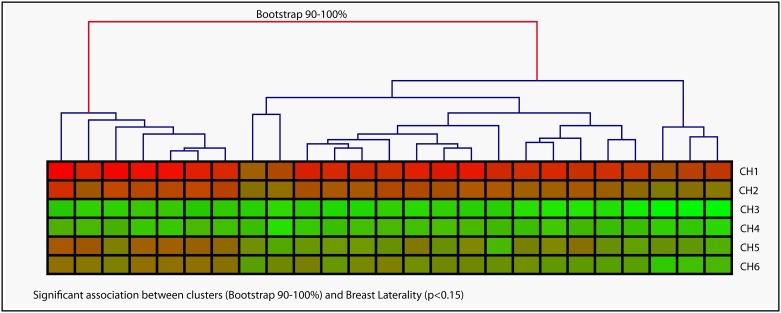
Cancer Hallmarks inferred from expression profiles correlate with breast laterality. In a different cohort of 25 breast tumors, Cancer Hallmarks were inferred from the expression profiles of 32 cancer related genes. CHs are represented in rows, tumors in columns. A color gradient from green to red is used to represent low to high values of CHs (from 5 to 52). By Unsupervised Hierarchical Cluster Analysis even though many clusters are formed, only two groups were established for bootstrapping 90–100% (shown by the red arm). By regression analyses a single association was found between clusters and the clinical variable BL.

When assessing whether the latter CH profiles -now inferred from expression profiles- segregated in a non-random manner, again we detected the formation of two groups by Unsupervised Hierarchical Cluster Analysis using a 90–100% bootstrap. The formation of these two groups encouraged us to perform further association analysis of the clusters with clinical variables. For subsequent Regression Analyses, p values less than 0.15 were considered relevant, and based on these criteria a unique association was observed between clusters and the clinical variable *BL* (p = 0.12) (Fig 6A). Again, when testing which CH had main participation in the cluster formation, multiple lineal regression analysis of clusters *vs*. CHs revealed that CH2, CH5 and CH6 were predictors of this clustering event (adjusted R^2^ = 0.67). When we checked whether this was observable when analyzing expression profiles (not converted to CH profiles), no clusters with 90–100% bootstrap were observed.

So far, experimental data inferred from methylation and expression analyses in different tumor cohorts were sustaining our BL hypothesis. To deeper confirm this, we searched for an *in silico* validation, by analyzing the public dataset from Oncomine Research Edition Platform (gene expression signatures). Filters were set for invasive breast cancer from the dataset “TCGA Breast”. Two hundred fourteen tumors presented anatomic location information (115 left sided and 99 right sided) and 28 of the 32 genes presented mean expression data (excluding RARB, DAPK1, MSH2 and DLC1 of Table 3). The expression data were similarly analyzed as data from experimental results: we inverted the expression mean values and converted them with the “translation matrix” to obtain a CH profile matrix. By unpaired Student test, we tested the means of left vs right sided tumors and surprisingly a significant difference was observed among them (p = 0.033, t = 2.45, R^2^ = 0.37).

Thus, these results validated our previous methylation-based observations in two manners: first, by detecting experimentally the same association between clusters and breast sides at a gene-expression level in a new cohort of fresh tumors; and secondly, by detecting the same difference *in silico*, when converting expression data of public databases into CH profiles.

## Discussion

Tumors evolve from benign to malignant cells by acquiring consecutive mutations over time. Similar to an evolution process which is modulated by natural selection pressures, during the tumorigenesis process the tumor acquires traits that confer survival advantages to the cells. Thereby, the acquired mutations which are found in the final tumor should have passed a selection filter and many of them are contributing in different levels to certain cancer pathways. Because most cellular processes involve multiple proteins functioning in a concerted and redundant manner, it is possible that mutations in different genes result in similar tumorigenic effects [17]. Thereby, when performing single-gene mutation studies, the complete tumor features can be missed. In contrast, when performing whole-genome-studies the overwhelming data can disturb the identification of specific tumor traits. When we clustered the studied genes in 6 selected cancer hallmarks and performed the statistical analyses on a higher organization level, we were able to notice the different hallmarks of right and left sided breast tumors.

In the current study we show for the first time, that breast carcinomas arising on different sides of the body present differential cancer traits inferred from methylation profiles. The validation by the expression analyses performed on a new experimental cohort of carcinomas and on public databases information supports our conclusions. Notably, numerous previous studies using single gene markers have failed in establishing a correlation between laterality and either methylation or expression levels. The fact that right and left sided breast tumors acquire differential tumor traits is reflected by our approach through the mean of transforming genetic and epigenetic data into cancer hallmarks.

Since the general understanding is that the breast tumors of different sides do not differ in behavior, clinical features, treatment response, etc., it becomes important to make clear whether no statistical artefacts are interfering in our results. A statistical artefact results from a bias in the collection, manipulation and/or measurement of the data. In our study, we can demonstrate that the bias sources have been avoided as discussed further on. *The collection biases* can be discarded since analyses were performed on two different and independent cohorts of breast tumors. The laterality of tumors in both cohorts was similar (left/right percentages: 56%/44% and 52%/48%, CI: 0.23–2.9, p = 0.01) and both cohorts revealed association of the cancer hallmark profiles with laterality. *The manipulation biases* can also be discarded based on the fact that the results have been observed on assays performed on two different molecules: DNA and RNA. That the observations based on the methylation assays (performed on DNA) and on the RealTime PCR expression assays (performed on RNA) converged into a similar association with side, shows that no manipulation of samples could be generating an artefact in the results. Furthermore, *measurement biases* can produce partial perceptions due to the used methods. We are aware of the potential artefacts that can appear when statistics is applied as a single approach. We therefore proposed questions which required different statistical analyses and who’s results converged and were in line with the same concept: cancer hallmark profiles differ among breast tumors of different sides. To answer the question whether tumors were distributed at random based on the cancer hallmark profiles, Unsupervised Hierarchical Cluster Analyses were performed. The formation of 2 significant clusters was observed both, from DNA methylation inferred data as from RNA expression analyses. It is worth to remark that the RNA expression observations were obtained in a complete different tumor cohort. To answer the question whether one specific cancer hallmark was contributing to the grouping of tumors, two different statistical analyses were applied: PCA and Multiple Regression Analyses. Both revealed that only some of the cancer hallmarks were relevant for the generation of the clusters. To answer if a clinical-pathological variables was associated with the clusters Simple Regression Analyses were performed and again, methylation and expression inferred data revealed association with side. Another way to confirm this association, was to analyze it backwards: partitioning the tumor sample in left and right sided, and by detecting a different distribution of the cancer hallmarks.

Taken all this together, we can asseverate that the observations are not statistical artefacts, in which case at least some of all the approaches (change of tumor cohort, change of molecule, change of statistic method) should have invalidated the rest of the observations.

And finally, the TCGA data support our findings, in an enhanced population of breast tumors (214 invasive ductal carcinomas). This has, to our consideration, the strongest statistical power to confirm and validate our results.

Among the different statistical analyses, CH6 (Invasion and Metastasis) appears as a key hallmark related to BL. Sixteen of the 43 studied genes do have a role in CH6, i.e. TIMP3, APC, ATM, PTEN, CD44, RASSF1A, CDH13, TP53, PAX5, THBS1, GATA5, DLC1, SFRP5, SFRP4, TWIST1 and RUNX3. The results evidence that in left sided tumors the CHM values for CH6 are higher than in right sided tumors. This challenging observation is difficult to interpret in clinical terms since deeper studies should evaluate the functional impact of high CH6 values on tumor cells behavior. We consider the consistent results on CH6, however, as a powerful support to postulate this cancer hallmark as the icon on which the BLH can be visualized.

Even though several studies present apparently discordances between laterality associations with clinical variables [18,19,20,21,22] Veltmaat *et al*. integrate these data in their recent review and discuss very interestingly that left-sided tumors are associated with more affected nodes despite the left-right asymmetry in overall node number. Therefore, metastasis to lymph nodes is proposed by them to be a lateralized disease feature, and may indicate left-right differences in tumor biology, e.g. greater metastatic potential of left-sided tumors and cancer, which is line with our observations.

Even though apparently symmetric, left and right sides of vertebrates’ bodies are different. Internal bilateral organs such as breast glands present differences in volume, structure and position. In tumorigenesis of paired organs, laterality has been described in breast [23], lung, testes [24] and kidney cancer [25]. Higher incidence of lung, ovarian and testicular cancer is found on the right side, whereas melanoma and breast cancer is approximately 10% and 5% respectively more likely to be diagnosed in the left side [26,27]. Speculations have been proposed to explain these diverse incidences, based on different organ sizes, handedness [28] and sun exposure [29]. However, the underlying reasons remain unknown.

In colon cancer, some works have discovered a tendency of different methylation profiles among left vs right sided adenomas [30,31]. Even though left and right sides of the colon present much more clear differences in their function and tissue environment, it is a proof of principle in line with our observations in the breast.

Only few studies exist about breast cancer laterality related to tumor behavior. Dane *et al*. informed in 2008 about an association of breast cancer laterality and lymph node metastasis in human patients [21]. Fatima *et al*. found that right sided breast tumors were associated with younger age and hormone receptor negativity [32]. Recently, Onibokun *et al*. informed that left sided predominance was significantly greater for high grade tumors and for hormone receptor negative tumors [33]. It should be interesting to analyze if and how the increment of the left/right ratio in higher grade tumors could be linked to our observations about the better predictive capacity of CH profiles in higher grade left tumors. Still, the regulation of these processes is far from clear.

Left-right sided asymmetries have been informed in gene expression during embryogenesis of the breast development [34]. Several growth factors such as Nodal, Lefty, FGF, HB-EGF and HGF as well as transcription factors (e.g. Pitx2, FoxA2) are considered candidates with overlapping functions in cancer and development laterality [34]. Recently, Robichaux *et al*. found that mammary glands in wild-type mice have differences in gene expression, and that these differences confer differential susceptibility to HER2-mediated effects on ductal epithelial growth and differentiation [35]. As communicated by Veltmaat *et al*. based on their observations in mice, it is becoming clear that each mammary gland has an individual identity since left-right asymmetries are acquired during embryonic mammary development [22]. Very interestingly, this latter publication reviews breast cancer left-right asymmetries, however some of them presenting contrasting data. Borisenkov *et al*. informed higher survival rates in right sided breast tumors of Russian patients [18], and on the contrary, Harveit *et al*. observed the same in left sided breast tumors of Norwegian patients [19].

Epigenetic alterations are proposed to occur linked to environmental variables [36]. Macro and micro-environment components can induce alterations in the regulated crosstalk which exists between DNA and histone marks, rendering a change in the gene transcriptome [37,38]. The epigenome, in contrast to the genome, is flexible, dynamic and reversible, being thereby a better candidate to respond faster to the environment. In this line, the breast gland is the specific micro-environment in which breast tumors develop. Tumor cells do not act in isolation, but rather subsist in a rich niche provided by resident fibroblasts, leukocytes, and extra-cellular matrix. Thereby, the tumor stroma is an integral part of cancer initiation, growth and progression. Our proposed breast laterality hypothesis sustains that left-right breast gland niches differ since their development. An example could be based on their different distance to the hart resulting in a distinct blood flow. The important role of inflammatory signaling in breast cancer suggests that a gradient in blood stream may influence tumor behavior [22]. We therefore propose that these left-right different micro-environments are the starting elements that during a tumorigenic process provoke distinct epigenetic signatures that are later on transformed into differential cancer hallmarks. We are aware that besides aberrant methylation, other alterations occur during tumorigenesis which can also contribute to the cancer hallmarks. Even though we have not evaluated what happens with the rest of genomic alterations in left and right breast tumors, we can affirm that at least the epigenetic contribution to cancer hallmarks, differs among sides. We are aware that our observations are solid but however partial, since we have included a selection of a few cancer related genes and organized them in a selection of several cancer hallmarks. Therefore, even though we were able to observe the differences among left and right sided tumors, further and deeper studies encompassing an extended genome analysis will give light to the laterality hypothesis.

A limitation of our study is that while in most of the cases it is true that methylation reduces the expression of the involved genes, there exist situations where this simple interpretation fails. In this study we performed methylation analyses on CpG sites located on promoters and first exons of cancer related genes, however this does not guarantee that they all regulate gene expression. It could be possible that in some cases more CpG sites are required for gene silencing, or where additional alterations, such as a complex crosstalk with histone modifications are needed for expression regulation. Moreover, genetic alterations such as copy number variations, translocations and point mutations can co-exist with epigenetic modifications. These effects have been underestimated in our work and could explain some discrepancies between methylation and expression data at a single-gene level. However, the impact of this limitation is reduced, given that both CH profiles -inferred from methylation and expression data- converged on the same association with BL. Therefore, gene expression data became interesting and powerful to re-evaluate the BLH. Our observations on CH profiles obtained from gene expression data are a strong support for the hypothesis given that: firstly, the experiments were performed on a completely new cohort of tumors; secondly, even though methylation events do not always imply gene silencing (as mentioned above), the laterality association of CHs persisted and raised from a complete different biologic observation. We consider this as a strong and powerful support for the BLH. Interestingly, the *in silico* analyses of gene expression signatures validated our experimental observations.

Our conclusions are relevant to the interpretation of breast cancer disease. We have demonstrated that epigenetic events can describe differential behavior of carcinomas in different breast sides, which should be informative for designing and better interpreting the results of experimental therapies. In fact, we propose that the use of similar approaches to the ones described in the current study may be useful for better understanding the potential link between heterogeneity in breast laterality with the malignant behavior and prognosis of breast cancer. Moreover, these results may guide the future design and execution of mechanistic experiments aimed at defining the genetic and epigenetic basis of side differences in breast cancer. Lastly, these results contribute with a new finding to the better understanding of breast tumor behavior, and can moreover serve as proof of principle for other bilateral cancers like lung, testes or kidney. In summary, our conclusions may influence future experimentations as well as diagnostic and therapeutic interventions in cancer types with left-right asymmetries.

## Supporting Information

S1 Fig Complete Translation Matrix. The Figure shows the gene-to-function Adjusted Participation Index (API) in a Translation Matrix, with values ranking their participation in the 6 CHs from 0 (no participation) to 3 (high participation). The matrix shows that among the 43 genes considered for our dataset, 24 presented associations to functions compatible with CH1, 7 with CH2, 20 with CH3, 8 with CH4, 15 with CH5, and 16 genes with CH6.
(TIF)

S1 File Supporting information. The file contains the tumor methylation profiles, the CH-methylation profiles and the clinical-pathological features of the cohort of 51 tumors and the tumor expression profiles, the CH-expression profiles and the clinical-pathological features of the different cohort of 25 tumors.
(XLSX)
