# Supplementary figures and images for: Asymmetric Cancer Hallmarks in Breast Tumors on Different Sides of the Body
Source: PLoS One. 2016 Jul 6;11(7):e0157416. doi: 10.1371/journal.pone.0157416 (PMC4934783; doi:10.1371/journal.pone.0157416)

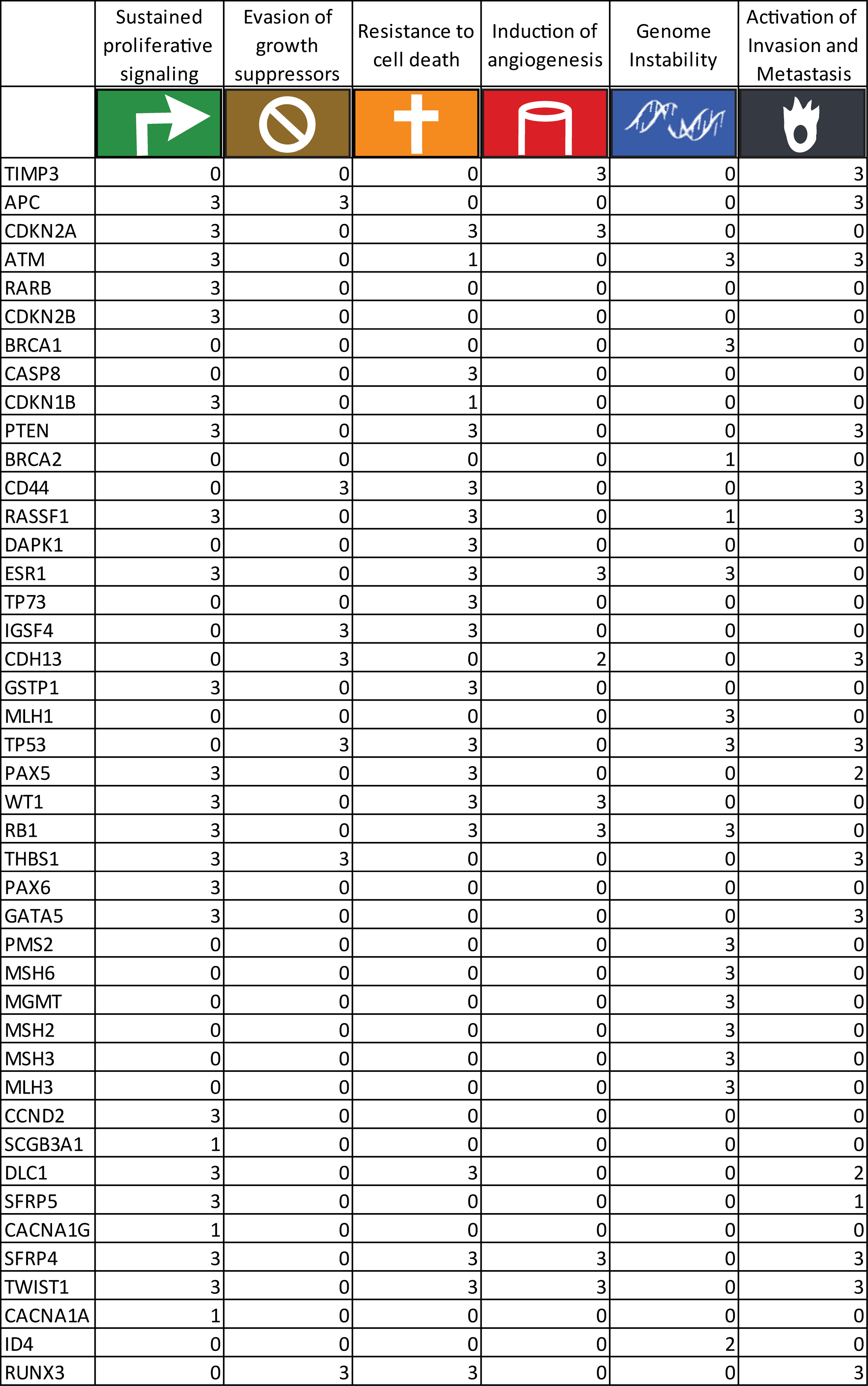

Supplement: S1 Fig — The Figure shows the gene-to-function Adjusted Participation Index (API) in a Translation Matrix, with values ranking their participation in the 6 CHs from 0 (no participation) to 3 (high participation). The matrix shows that among the 43 genes considered for our dataset, 24 presented associations to functions compatible with CH1, 7 with CH2, 20 with CH3, 8 with CH4, 15 with CH5, and 16 genes with CH6. (TIF) [file pone.0157416.s001.tif]
